# Retinoic Acid Receptor β Loss in Hepatocytes Increases Steatosis and Elevates the Integrated Stress Response in Alcohol-Associated Liver Disease

**DOI:** 10.3390/ijms241512035

**Published:** 2023-07-27

**Authors:** Marta Melis, Steven E. Trasino, Xiao-Han Tang, Andrew Rappa, Tuo Zhang, Lihui Qin, Lorraine J. Gudas

**Affiliations:** 1 Department of Pharmacology, Weill Cornell Medical College of Cornell University, New York, NY 10065, USA; mam2185@med.cornell.edu (M.M.);; 2 Nutrition Program, Hunter College, City University of New York, New York, NY 10065, USA; 3 Genomics Resources Core Facility, Weill Cornell Medical College of Cornell University, New York, NY 10065, USA; 4 Division of Anatomic Pathology, New York Presbyterian Hospital, Department of Pathology and Laboratory Medicine, Weill Cornell Medical College of Cornell University, New York, NY 10065, USA

**Keywords:** vitamin A, oxidative stress, nuclear receptor, alcohol toxicity, ATF4

## Abstract

In alcohol-associated liver disease (ALD), hepatic reductions in vitamin A and perturbations in vitamin A metabolism are common. However, the roles that the vitamin A receptors, termed retinoic acid receptors (RARs), may have in preventing the pathophysiology of ALD remains unclear. Our prior data indicate that a RARβ agonist limits the pathology of alcohol-related liver disease. Thus, we generated liver-specific AlbCre–RARβ knockout (BKO) mice and compared them to wild type (WT) mice in an early ALD model. Both strains showed similar blood ethanol concentrations and ETOH-metabolizing enzymes. However, the livers of pair-fed-BKO and ETOH-BKO mice developed higher levels of steatosis and triglycerides than pair-fed-WT and ETOH-WT mice. The increased hepatic steatosis observed in the pair-fed-BKO and ETOH-BKO mice was associated with higher lipid synthesis/trafficking transcripts and lower beta-oxidation transcripts. ETOH-BKO mice also exhibited a higher integrated stress response (ISR) signature, including higher transcript and protein levels of ATF4 and its target, 4-EBP1. In human hepatocytes (HepG2) that lack RARβ (RARβ-KO), ETOH treatments resulted in greater reactive oxygen species compared to their parental cells. Notably, even without ETOH, ATF4 and 4-EBP1 protein levels were higher in the RARβ-KO cells than in their parental cells. These 4-EBP1 increases were greatly attenuated in cultured ATF4-deficient and RARβ/ATF4-deficient HepG2, suggesting that RARβ is a crucial negative regulator of 4-EBP1 through ATF4 in cultured hepatocytes. Here, we identify RARβ as a negative regulator of lipid metabolism and cellular stress in ALD.

## 1. Introduction

Alcohol-associated liver disease (ALD) is one of the main, chronic liver disorders, and together with metabolic dysfunction-associated steatotic liver disease and steatohepatitis (MASLD/MASH, previously termed NAFLD/NASH), ALD is a major risk factor for both liver cancer and death [1,2]. The liver is the key organ that metabolizes ethanol (ETOH), mainly via alcohol dehydrogenases [3], and, at high levels of ethanol intake, through cytochrome P450 2E1 (CYP2E1), which is localized in the pericentral region of the liver [3,4]. ETOH disrupts cellular homeostasis through multiple mechanisms, including the generation of reactive oxygen species (ROS) [3], an increased demand for processing protein folding that causes endoplasmic reticulum stress and the unfolded protein response [5], and increased mitochondrial dysfunction [6], that can result in the integrated stress response (ISR) with eventual cell death [7].

A large body of data have shown that chronic ETOH intake decreases hepatic levels of vitamin A (VA, retinol) and some of its metabolites, collectively known as retinoids [8,9], but the reasons for this decrease remain unclear. Vitamin A is an essential micronutrient that is acquired through the diet and is primarily stored as retinyl esters in hepatic stellate cells [10,11]. The active metabolite of VA is retinoic acid (RA), which is the endogenous agonist of the RA receptors (RARs) α, β, and γ [12]. These RARs bind DNA as RAR/RXR (retinoid X receptor) heterodimers in the absence of RA to inhibit the gene transcription of their specific target genes; the agonist RA results in a conformational change in the RAR/RXR complex, allowing the transcriptional activation of their primary target genes to take place [13]. These RARs are important regulators of development, immunity, and metabolic functions [12]. While a reduction in VA levels in the liver is associated with human ALD [8,14] and the treatment of ETOH-fed rodents with RA mitigates ALD [15], the functions and roles of RARs in ALD are unclear.

Our group showed that wild type (WT) mice fed a high-fat diet (HFD), recapitulating MASLD, or treated with ethanol (ETOH), mimicking aspects of ALD, exhibit reduced hepatic retinol and retinyl esters and increased steatosis, cellular stress, and inflammation [16,17,18]. In both models, treatment of WT mice with a selective RARβ2 agonist (with RARβ2 being the most abundant RARβ isotype), AC261066, limited hepatic steatosis, cellular stress, and inflammation compared to mice not given AC261066 [16,17,18,19]. We also showed in a murine model that treatment with AC261066 limited MASLD, whereas treatment with the RARα agonist AM80 exacerbated steatosis and inflammation [18]. Furthermore, RARβ was required for AC261066 to reduce the levels of lipid droplets in cultured hepatocytes, indicating that AC261066 likely acts via RARβ in these cells [19]. These data led us to hypothesize that RARβ is a key transcription factor that protects the liver from ETOH-induced stress.

In this study, we dissected the mechanisms by which RARβ protects the liver from ETOH-induced stress. We used the Lieber–DeCarli liquid diet model to mimic the aspects of early ALD in C57Bl/6 wild type (WT) and AlbCre; RARβ^fl/fl^ (liver-specific RARβ knockout (BKO)) mice. We showed that ETOH-BKO mice exhibited more severe ALD compared to WT mice, suggesting that RARβ limits ETOH-induced stress. We further obtained similar results in RARβ-deficient cultured hepatocytes. This research highlights a previously unrecognized causal role for RARβ in the attenuation of ALD pathogenesis.

## 2. Results

### 2.1. RARβ Protects Hepatocytes from Alcohol-Associated Steatosis

To understand the functions underlying RARβ in hepatocytes we generated liver-specific (AlbCre) RARβ knockout mice (hereafter referred to as BKO). We validated the BKO mouse line with Southern blotting and detected a 5.5 kilobase band in the liver (Supplementary Figure S1A–C), indicating that the RARβ gene has been cut by Cre [20]. Using qRT-PCR, we also measured an 11-fold (±0.0004; *p* = 0.01) decrease in RARβ mRNA in livers of the BKO mice compared to the wild type (WT) mice (Supplementary Figure S1D).

The mice on the isocaloric control pair-fed (PF) and ETOH diets maintained similar body weights and consumed similar amounts of their liquid diet throughout these treatments (Supplementary Figure S2A,B). The ETOH-WT and ETOH-BKO groups displayed comparable ETOH blood levels (Supplementary Figure S2C) and levels of the CYP2E1 protein, one of the main ETOH-metabolizing enzymes (Supplementary Figure S2D,E). Moreover, ETOH caused similar increases in the levels of TNFα in the WT and BKO mice groups (Supplementary Figure S3).

**Figure 1:**
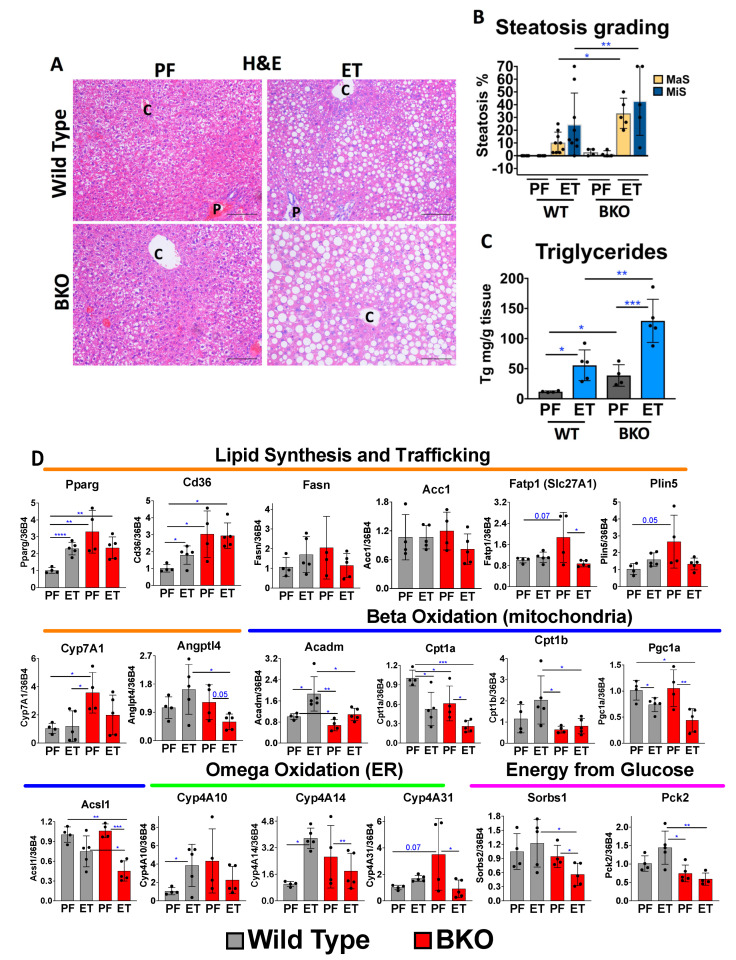
Phenotype of the Liber DeCarli ethanol-fed mice. (**A**) Hematoxylin and eosin (H&E) staining of representative liver sections of pair-fed (PF) and ethanol (ET) treatments in wild-type and AlbCre–RARβ knockout (BKO) mice. “C” denotes the hepatic central vein and “P” represents the portal vein, respectively. (**B**) Steatosis grading performed by a liver pathologist blinded to the identity of the treatments. The treatments are indicated as either the pair-fed (PF) diet (or control diet) or the ETOH diet (ET). MaS indicates macrosteatosis and MiS indicates microsteatosis, respectively. (**C**) Quantitative measurements for the levels of hepatic triglycerides performed with the Folch method (Materials and Methods). The levels of triglycerides (Tg) are expressed as Tg per milligram of tissue. The data are represented as mean ± standard deviation (SD). Mice used for each group: WT (PF = 6; ET = 8, respectively) and BKO (PF = 4; ET = 6, respectively). (**D**) mRNA levels of the genes grouped under different categories: lipid synthesis and trafficking under the orange line, beta-oxidation occurring in the mitochondria under the blue line, omega oxidation occurring in the endoplasmic reticulum (ER) under the green line, and energy released from glucose under the pink line in the wild-type (gray bars) and BKO mice (red bars) fed a pair-fed (PF) diet and an ETOH diet (ET). WT (PF = 4; ET = 5, respectively). BKO (PF = 4; ET = 5, respectively). The data are represented as mean ± standard deviation (SD). Scale bar = 100 μm. * = *p* < 0.05; ** = *p* < 0.01; *** = *p* < 0.001; **** = *p* < 0.0001.

We then compared our WT versus BKO mice to determine whether the BKO mice displayed a more ETOH-associated pathology. Histopathologic evaluations revealed that the ETOH-BKO livers exhibited increased macro- and micro-steatosis levels, mainly around the mid-lobular and central vein areas of the liver, compared to the ETOH-WT mice (Figure 1A,B). Similarly, using a biochemical method for triglyceride extraction, we found that the ETOH-BKO livers showed a 2.3-fold (±1.5; *p* = 0.02) increase compared to the ETOH-WT livers (Figure 1C). The BKO mice fed the pair-fed (PF) liquid diet also showed increased triglyceride levels compared to the WT mice on the same diet (Figure 1C), indicating that RARβ-deficient hepatocytes are more susceptible to steatosis both with and without ETOH.

To identify the signaling pathways enriched in this model of ALD, we performed RNA-seq analysis of the livers of the WT mice fed the ETOH-based diet versus the PF diet. We identified 13,274 differentially expressed transcripts (Supplementary File S1), of which 1011 of those showed a fold change ≥ 1.5. Predictably, we found increases in the levels of lipid-associated transcripts (Supplementary File S1) among the transcripts with the highest fold changes in the ETOH-WT mice compared to the PF-WT mice. To verify the levels of these lipid-associated mRNAs between the WT and BKO mice, we performed qRT-PCR. The mRNA levels of the key genes involved in lipid uptake and processing, e.g., Pparg, Cd36, Fatp1, and Plin5, were found to be higher in the PF-BKO mice compared to the PF-WT mice (Figure 1D), reflecting the increased triglyceride levels we previously observed in the BKO mice on the PF diet (Figure 1A–C). However, ETOH feeding did not further elevate the levels of these transcripts in the BKO mice. Key lipid synthesis-associated transcripts, which include Fasn and Acc1, did not show any significant changes across the experimental groups. However, the expression of Angptl4, which can promote triglyceride clearance in hepatocytes [21], showed a decrease of over 2-fold in the ETOH-BKO mice compared to the ETOH-WT mice. Meanwhile, the transcript levels of Cyp7A1, a key gene for bile acid synthesis, were increased in the PF-BKO group compared to the PF-WT group. Previous reports have indicated that retinoic acid treatment decreases the mRNA levels of Cyp7A1 in hepatocytes [22], thus suggesting that the Cyp7A1 increases observed in the PF-BKO mice could be mediated by RARβ [22].

In addition to the increased levels of lipid synthesis-associated mRNAs observed in the PF-BKO group, we detected evidence for decreased lipid beta-oxidation, as shown by the lower Acadm and Cpt1a mRNA levels in the PF-BKO vs. WT mice. We also discovered a more dramatic decrease in the levels of beta-oxidation in the ETOH-BKO mice compared to the ETOH-WT mice, as indicated by the decreases in the Acadm, Cpt1b, Pgc1a, and Acsl1 mRNAs (Figure 1D). To overcome impaired beta-oxidation, compensatory omega oxidation could occur [23]. Our data showed that ETOH increased the transcript levels of the genes associated with omega oxidation in the WT mice, but not in the BKO mice. We also measured the mRNA levels of the genes implicated in glucose transport (Sorbs1) and gluconeogenesis (Pck2) and showed that these transcripts were decreased in the ETOH-BKO group. Collectively, decreased beta-oxidation, along with simultaneous lipid synthesis, could result in higher steatosis levels in the BKO mice even without ETOH treatment.

### 2.2. RARβ Limits the ETOH-Associated Integrated Stress Response (ISR) in Mice

**Figure 2:**
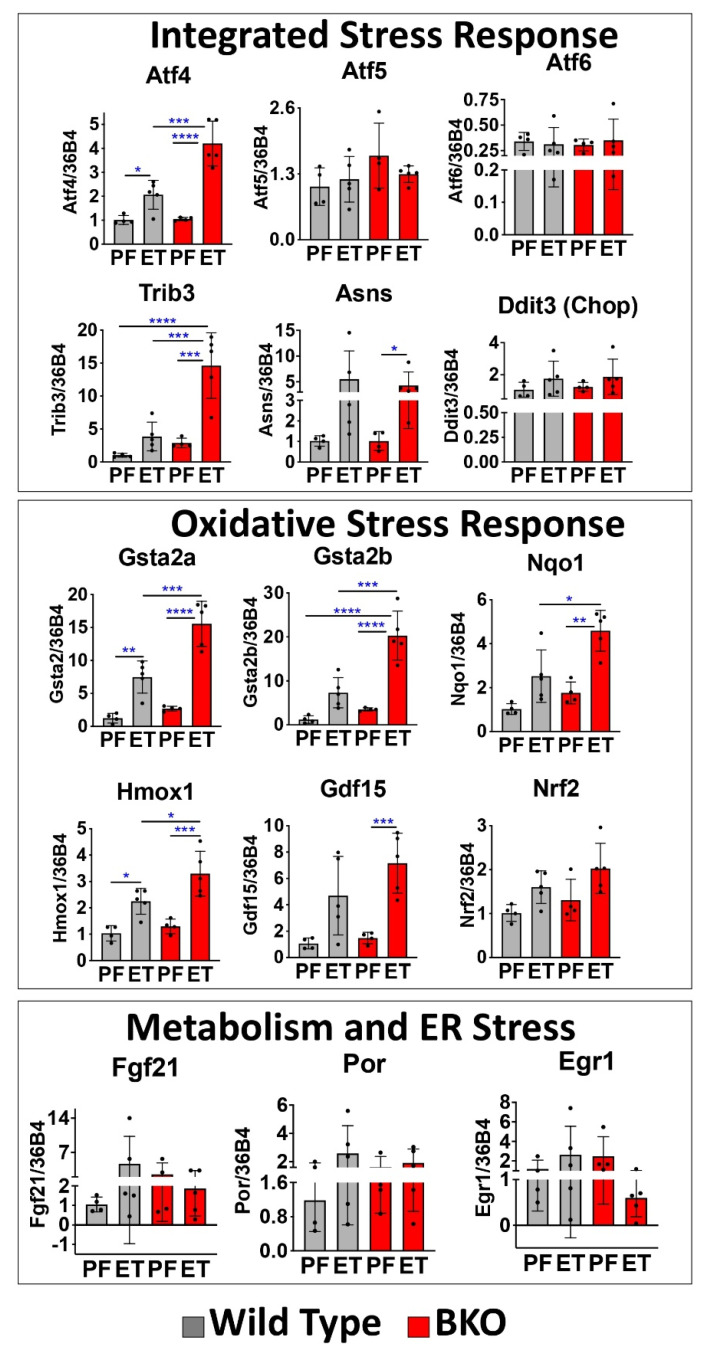
Genes associated with oxidative Stress. mRNA levels of the genes associated with the integrated stress response, the oxidative stress response, metabolism, and endoplasmic reticulum (ER) stress in the wild type (grey bars) and BKO mice (red bars) fed a pair-fed (PF) diet and an ETOH diet (ET). WT (PF = 4; ET = 5, respectively). BKO (PF = 4; ET = 5, respectively). The data are represented as mean ± standard deviation (SD). * = *p* < 0.05; ** = *p* < 0.01; *** = *p* < 0.001; **** = *p* < 0.0001.

We also used the pathway analysis web resource, Enrichr (maayanlab.cloud/Enrichr/) to analyze the RNA-seq data we obtained and found that oxidative stress-related pathways were among the top ten pathways (Supplementary File S2) observed from four out of seven gene enrichment databases. Our RNA-seq analysis revealed the major pathways and key transcripts associated with the hepatic oxidative stress response (i.e., Gsta2, Nqo1, Gdf15, and Ddit3 (Chop)) [24]), with some of these genes (Gdf15 and Ddit3) being indicative of a more severe and prolonged stress known as the integrated stress response (ISR) [25,26] (Supplementary File S2). We further investigated the oxidative stress response and the ISR signature key transcripts using qRT-PCR in the WT and BKO livers (Figure 2). Compared with the ETOH-WT group, the ETOH-BKO livers showed increased mRNA levels of the ISR-associated genes Atf4 and Trib3 (Figure 2). Other mRNAs that are part of the Atf4 ISR signature and the oxidative stress response [27], including Asns and Gdf15 [25,28,29], were also increased in the livers of the ETOH-BKO and ETOH-WT groups compared to the PF groups (Figure 2). We measured the hepatic mRNA levels of Atf6, an endoplasmic reticulum stress marker, but detected no significant changes across the experimental groups (Figure 2). Similarly, we observed no significant changes in a key ATF6-associated chaperone protein, BIP/GRP78 (Supplementary Figure S4) [30], indicating that unlike ATF4 and its network, ATF6 likely does not play a major role in our model of ALD. We further investigated a third arm of the classic ER stress response, IRE1α–XBP1 splicing [5], and found no spliced Xbp1 in our model (Supplementary Figure S5). Together, these mRNA data indicate that the loss of RARβ in hepatocyte cells exacerbates the ETOH-induced activation of oxidative stress and the ISR/ATF4 gene signaling pathway in the liver.

Next, we discovered that transcripts that are part of the oxidative stress response and function as antioxidants, including Gsta2a and Gsta2b, as well as Nqo1 and Hmox1 (Figure 2), were increased in the ETOH-BKO mice compared to the ETOH-WT mice, suggesting the production of a greater response to cellular stress in the ETOH-BKO mice. Nqo1 and Hmox1 are target genes of Nrf2, a key transcription factor that regulates the cellular antioxidant defense [6]. However, Nrf2 mRNA levels were only minimally increased in the ETOH-treated mice (Figure 2). Genes that have previously been linked to different aspects of the oxidative stress response, including Atf5, Fgf21, Egr1, and Por, showed no transcript increases in the livers from the ETOH-BKO vs. ETOH-WT mice, suggesting that these genes are not driving our model of ALD pathology (Figure 2).

**Figure 3:**
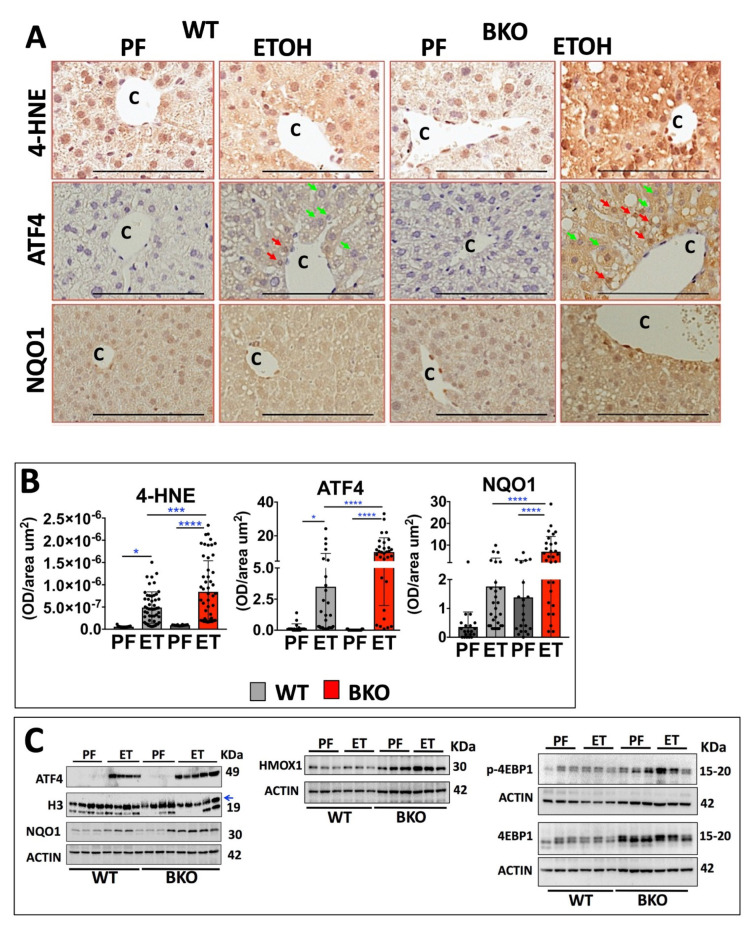
Increased oxidative stress and the ATF4 network in RARβ-deficient hepatocytes compared to wild type mice. (**A**) Immunohistochemistry of the oxidative stress marker 4-hydroxynonenal (4-HNE), ATF4, and NQO1, in the livers of the AlbCre–RARβ knockout (BKO) and wild type (WT) mice. We performed immunostaining in representative liver sections of pair-fed (PF) mice (WT, n = 4; BKO, n = 4) and ETOH-fed mice (WT, n = 5; BKO, n = 5). “C” denotes the hepatic central vein and “P” designates the portal vein, respectively. The red arrows denote ATF4-positive nuclei and green arrows denote ATF4-negative nuclei. (**B**) Quantification of the immunostainings for 4-HNE, ATF4, and NQO1. (**C**) Western blotting of the protein expression of ATF4, NQO1, HMOX1, phosphorylated 4-EBP1 (T37/46), and total 4-EBP1. The blue arrow denotes the specific size band for H3. The data are represented as mean ± standard deviation (SD). Scale bar = 100 μm. * *p* < 0.05; *** = *p* < 0.001; **** *p* < 0.0001.

We next performed immunostaining to measure 4-hydroxynonenal (4-HNE), a lipid peroxidation product and widely used marker of oxidative stress [31]. The ETOH-BKO livers exhibited increased 4-HNE levels (72%, *p* = 0.001) compared to that of the ETOH-WT livers (Figure 3A,B; Supplementary Figure S6). Similarly, the hepatic protein levels of ATF4 and NQO1 were markedly higher in the ETOH-BKO mice than in the ETOH-WT mice, as demonstrated in the high and low magnification images that were obtained following the immunostaining procedure (Figure 3A,B; Supplementary Figure S6). The red arrows point to nuclear ATF4 positivity, especially in the hepatocytes of the ETOH-BKO mice. This is a strong indication of increased ATF4 activation (Figure 3A). Next, with Western blotting, we found similar increases in the levels of the ATF4, NQO1, and HMOX1 proteins (Figure 3A–C). The higher transcript levels of the key genes in the Atf4 pathway, such as Trib3 and Asns, which were observed in the ETOH-BKO livers (Figure 2 and Figure 3) led us to measure 4-EBP1 (EIF4EBP1), as it is a critical ATF4 gene target that is implicated in protein synthesis, metabolic and stress adaptations [29], and cancer [32]. We found increases in the phosphorylated form of 4-EBP1 (T37/46) in the ETOH-BKO mice, whereas we found increases in the levels of the total 4-EBP1 protein in both the PF- and ETOH-BKO livers vs. the WT livers (Figure 3C). Taken together, these findings support a model in which RARβ limits the levels of ATF4 and 4-EBP1 in hepatocytes.

### 2.3. RARβ Knockout-Cultured Hepatocytes also Show Increased Reactive Oxygen Species Generation

**Figure 4:**
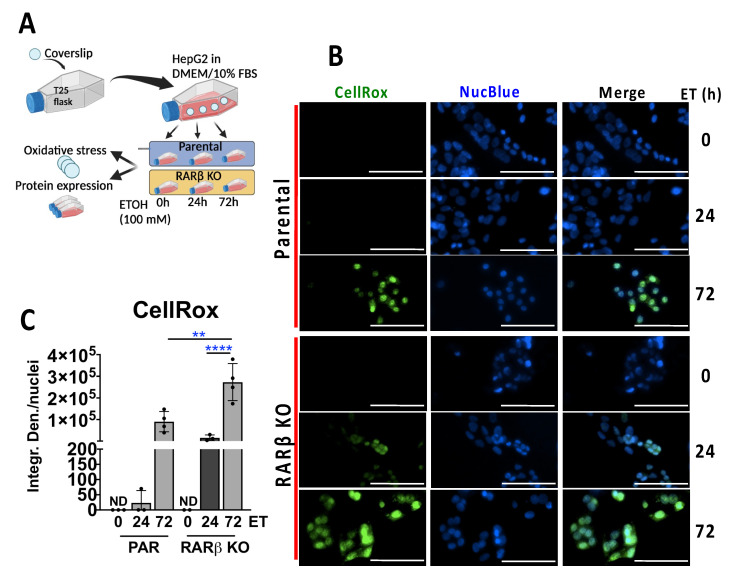
ETOH-induced oxidative Stress in RARβ knockout human hepatocytes. (**A**) Experimental strategy in RARβ knockout (KO) previously generated using Crispr/Cas9 technology with HepG2 cells (Ref. [19]). For the ETOH treatments, we plated cells in T25 flasks in which we previously placed sterile glass coverslips and allowed the cells to attach for 24 h in DMEM supplemented with 10% FBS (Supplementary Materials and Methods). The use of T25 flasks provided a reliable method to avoid ETOH evaporation and glass coverslips in the T25 flasks could be removed following the end of these experiments to measure the levels of oxidative stress in living cells. The remaining cells in the T25 flasks were scraped for Western blotting. ETOH treatments (100 mM) were first performed for 24 and 72 h and then compared with untreated cells, indicated as 0 h. (**B**) To assess the levels of oxidative stress in the experimental groups, we stained the cells for 40 min with 2.5 μM of CellRox (green), whereas for the nuclei, we used NucBlue (blue). (**C**) Quantification of the CellRox staining. CellRox staining expressed as integrated density (Integr. Den.), normalized for the number of nuclei per field. Scale bar = 100 μm. ** = *p* < 0.01; **** = *p* < 0.0001.

To investigate the effects of ETOH on ATF4 in cultured hepatocytes, we used the human hepatoma cell line HepG2, along with the normal mouse hepatocyte cell line AML12, to ensure that the ETOH-induced effects were not unique to the neoplastically transformed phenotype of HepG2 cells. Additionally, in order to determine whether hepatocytes play a direct role in the increased susceptibility of the BKO mice to ETOH-induced stress, we generated a Crispr/Cas9 RARβ knockout HepG2 cell line [19]. After 24 h and 72 h ETOH treatments (Figure 4A), we stained living cells with the reactive oxygen species (ROS) dye CellRox and found that ROS levels caused by the ETOH treatments increased over time in both the RARβ-KO and parental cells (Figure 4B,C). However, the RARβ-KO cells displayed higher ROS levels compared to their parental cells after both 24 h and 72 h of ETOH (Figure 4B,C). Thus, compared to the parental cells, RARβ-deficient hepatocytes exhibit more rapid production and higher levels of ROS upon ETOH treatment.

### 2.4. ATF4 Is Increased in the RARβ Knockout-Cultured Hepatocytes

**Figure 5:**
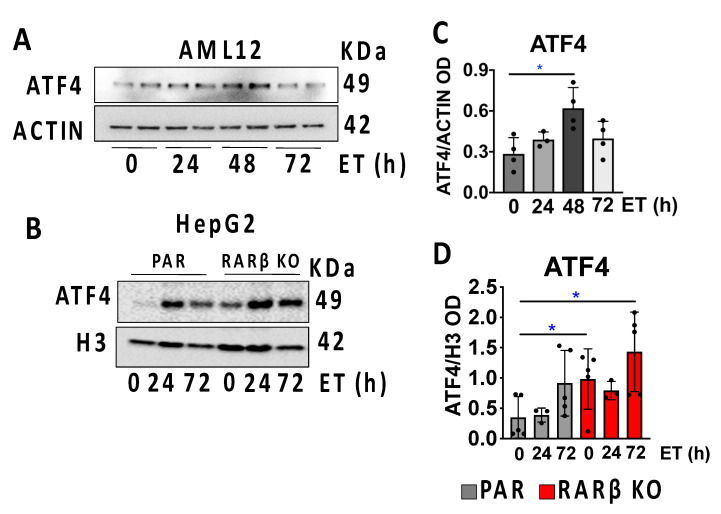
ATF4 increases in RARβ knockout human and mouse hepatocytes. (**A**) ATF4 Western blotting in the mouse hepatocyte cell line, AML12, used as normal hepatocytes of reference after ETOH treatments. Representation of one of the three experiments performed. Treatments were performed for 24, 48, and 72 h, respectively, and were compared to no ETOH treatment group (0 h). (**B**) Representative Western blotting of ATF4 in the parental (PAR) and RARβ KO HepG2 cells. (**C**) Relative ATF4 quantification in all experiments performed using AML12 cells calculated as the ratio between the optical density (OD) of the protein of interest and that of the loading control (actin). (**D**) Relative quantification in all experiments performed using HepG2 cells calculated as the ratio between the optical density (OD) of the protein of interest and that of the loading control (actin). The data are represented as mean ± standard deviation (SD). * *p* < 0.05.

Ethanol caused ATF4 increases in both the AML12 (Figure 5A,C) and HepG2 cells (Figure 5B,D). Next, we noted that even RARβ-KO cells cultured without ETOH treatment displayed a 1.5-fold increase in the ATF4 protein compared to the parental HepG2 cells, indicating that lack of RARβ increases ATF4 even under routine culture conditions, an indication of increased susceptibility to cellular stress.

### 2.5. RARβ Is a Negative Regulator of ATF4 but Not 4-EBP1 in Cultured Cells

**Figure 6:**
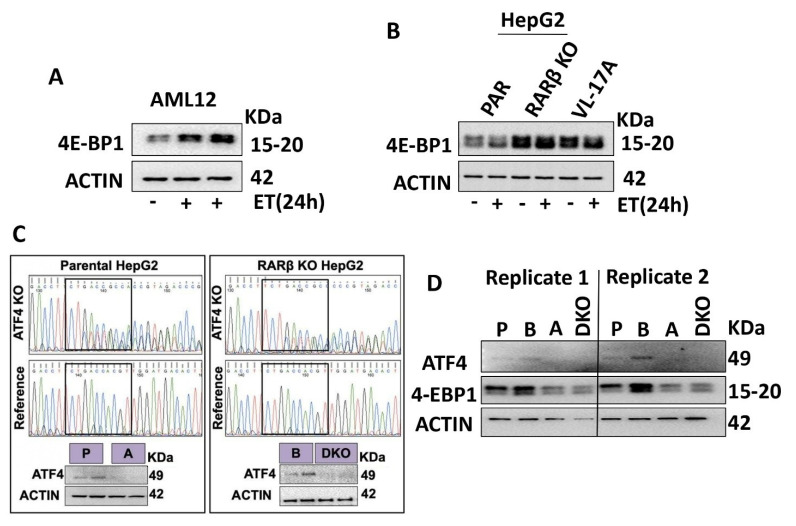
4-EBP1 increases in RARβ knockout human hepatocytes depend on ATF4. (**A**) 4-EBP1 Western blotting in the mouse hepatocyte cell line, AML12, used as normal hepatocytes of reference after ETOH (ET) treatments for 24 h. (**B**) 4-EBP1 Western blotting in the parental (PAR) and RARβ-KO HepG2 cells, and VL-17A cells (HepG2 cells that overexpress CYP2E1 used here as a reference) treated with ETOH for 24 h. (**C**) Visualization and verification via Western blotting of ATF4 elimination following CRISPR/Cas9 technology application. (**D**) Two replicate experiments measuring the protein levels of ATF4 and 4-EBP1 in the parental (P), RARβ-KO (B), ATF4 KO (A), and RARβ-ATF4 double knockout (DKO) HepG2 cell lines.

We next asked whether the ATF4 increases observed in the RARβ-KO HepG2 cells were associated with increases in its target, 4-EBP1, thus recapitulating the results obtained from the PF-BKO and ETOH-BKO mice. Again, we found that ETOH treatment caused similar 4-EBP1 increases in both AML12 and HepG2 cells (Figure 6A,B). Consistent with the ATF4 increases observed in the RARβ-KO HepG2 cells, 4-EBP1 protein levels were also increased in the RARβ-KO cells. To further ascertain whether the 4-EBP1 increases observed in the RARβ-deficient hepatocytes were directly dependent on ATF4, we generated an ATF4 KO cell line in both the parental and RARβ-KO lines (Figure 6C). Comparing the 4-EBP1 levels in the ATF4 KO and the RARβ-KO cells; the 4-EBP1 levels in ATF4 KO cells were greatly decreased. These results show that 4-EBP1 levels in cultured cells are primarily regulated by ATF4.

## 3. Discussion

Marked reductions in hepatic retinoids occur in human and animal models of chronic alcohol abuse [14,33]. Here we demonstrated that hepatocyte loss of one of the effectors of retinoid signaling, RARβ, is associated with a greater ETOH-induced level of hepatic steatosis and oxidative stress.

### 3.1. ETOH-Fed BKO Mice Develop Greater Hepatic Steatosis Than Wild Type Mice

Hepatic steatosis is one of the first histological changes that occur in the livers of mice fed an ETOH-diet, such as the Lieber–DeCarli diet [34]. Here, we showed that BKO mice exhibited higher levels of steatosis compared to the WT mice on both a PF diet and after ETOH treatment (Figure 1). The increased steatosis observed in the livers of the BKO mice after ETOH treatment is consistent with our recently published data indicating that RARβ-KO HepG2 cells treated with oleate and palmitate show dramatic increases in the numbers of lipid droplets compared to HepG2 parental cells [19]. Thus, our current data reported here underscores the role of RARβ in limiting steatosis in two different models, one caused by high fat intake [19], and one by ETOH [17].

We do not know whether RARβ limits steatosis by repressing specific genes in hepatocytes in the absence of the endogenous agonist RA, or if RA is required for either transcriptional activation or repression for RARβ to limit steatosis. Experiments in which the BKO mice are placed on a vitamin A-deficient diet and then given ETOH could potentially answer this question, but such experiments are complicated by the effects of vitamin A depletion on other organs in the body. In a recent publication on a nuclear receptor related to the RARs, LXR (liver X receptor), a set of LXR target genes in the liver was identified that show direct ligand-dependent transcriptional repression; these genes exhibited an increased expression in the LXR knockout mice, but they also displayed lower expression levels in response to an LXR agonist [35]. It is possible that RARβ can regulate a subset of target genes in a similar manner.

The increased steatosis in the BKO mice compared to the WT mice on a pair-fed diet was consistent with lipid synthesis and transport transcript increases, and surprisingly with the deficient lipid beta-oxidation (Figure 1). In a recent publication, hepatic stellate cell-specific Cpt1a knockout mice showed dramatic hepatic steatosis increases when fed with both control and high-fat diets [36], indicating how beta-oxidation is key to the overall lipid metabolism of the liver.

Other researchers have reported that the actions of the RARs can limit the hepatosteatosis associated with obesity. Li et al. [37] demonstrated that RARβ promotes fatty acid oxidation in a mouse model of obesity-associated metabolic disease, but while these authors invoked increases in FGF21 as the major mechanism by which RARβ acted in this murine model, we did not detect lower FGF21 mRNA levels in the BKO livers compared to the WT livers (Figure 2). Expression of a dominant negative RARα construct, which blocks the actions of all three RARs in hepatocytes, also resulted in steatohepatitis and liver tumors [38]—a dramatic result. However, in this model, the effects of each RAR were not able to be determined since the actions of all three RARs were blocked by the dominant negative construct. In a RARα knockout (AlbCre) mouse model fed an obesogenic diet, the increased hepatosteatosis, relative to that observed in the RARα fl/fl mice used as controls, was prevented by the treatment with the RAR pan-agonist, all-trans retinoic acid (RA) [39], indicating that RA can act via RARs other than RARα to limit hepatosteatosis. However, in this report, no RARα selective agonist was assessed. Collectively, our data on RARβ and previous research by others on RARα indicate that RARs are protective against steatosis and could be exploited therapeutically to limit the onset of liver disorders characterized by a dysregulation of lipid metabolism. However, we need to further dissect the mechanistic links between the RARs and the various lipogenic metabolic pathways in the liver.

### 3.2. Lack of RARβ Causes Greater Oxidative Stress and the Activation of the ATF4/Integrated Stress Response during ETOH Treatment

One of the unexpected signatures emerging from the ETOH-BKO hepatocytes compared to WT is the activated ATF4 network, which includes the increased mRNA and protein levels of the transcription factor ATF4 and the mRNA levels of several of its key target genes, such as Asns and Trib3 [28,29]. Both are ‘classic’ ATF4 target genes [40]. Previous research demonstrated that ATF4 knockout (AlbCre) in the livers of mice on a 4 week-long Lieber–DeCarli diet resulted in resistance to the development of hepatosteatosis and among the key mechanisms by which ATF4 increased steatosis were increased lipogenesis, a lipid uptake gene signature, and increased AMPK activity [41]. In our RNA-seq analysis, oxidative stress-associated pathways, rather than lipid metabolism-associated pathways, were predominant in the gene signature.

Although there is evidence in that obese mice and ETOH-treated mice display a reduced level of oxidative stress after treatment with RA (a pan RAR agonist) [42] or a RARβ2 agonist (AC261066) [17], we did not understand which molecular pathways participated in the stress response to ETOH. Our data, presented here, support a previously unrecognized role for RARβ in negatively regulating ETOH-induced cellular stress. Thus, when RARβ is absent, ATF4 mRNA and protein expression levels are higher (Figure 2 and Figure 3). ATF4 is often primarily regulated at the level of protein translation [24], but in the ETOH-BKO livers, ATF4 mRNA is higher compared to the ETOH-WT livers (Figure 2). Our data suggest that there is transcriptional activation of the ATF4 gene in the absence of RARβ, raising the possibility that RARβ either regulates ATF4 directly or acts indirectly as an ATF4 regulatory transcription factor. Presently, no research has been conducted to determine these mechanisms. We also recently reported that ATF4 mRNA levels are much higher in human clear cell renal cell carcinoma patient specimens than in normal kidney samples [28].

Notably, one of the ATF4 best-described target genes is EIF4EBP1, which encodes 4-EBP1 [29], a protein required to decrease mRNA translation in physiologic states [43]. Persistent, chronic 4-EBP1 overexpression is associated with multiple types of cancer [32]. Although we show that both mice and cultured hepatocytes with RARβ deficiency display large ATF4 and 4-EBP1 increases, our RARβ-ATF4 double knockout cell cultures did not show increases in 4-EBP1 (Figure 6). These data implicate RARβ as a key tumor suppressor in the liver and require further research to determine how RARβ and ATF4 interact.

### 3.3. Cyp2E1 Is Not Required for the More Severe ALD and Increased ISR Network in the BKO Liver

An increased CYP2E1 in ALD is associated with cellular stress and the integrated stress response (ISR) [44], and is critical for the pathology of ALD in mice and humans [5]. Our data show that even though the ETOH-BKO mice display higher steatosis levels and greater expression of the ISR network compared to the ETOH-WT mice, their blood ETOH levels and liver CYP2E1 protein levels are not higher (Supplementary Figure S2), suggesting that, in part, RARβ regulates cellular stress independently from CYP2E1.

In summary, our research advances our current understanding of both the pathophysiology of ALD and the role of the retinoic acid signaling pathway in liver physiology, with RARβ as a key negative regulator of steatosis, cellular stress, and the integrated stress response via ATF4 (summarized in the graphic abstract). Our results identify previously undetected links between the retinoic acid and the integrated stress response signaling pathways, and provide a deeper understanding of the pathophysiology of ALD. This knowledge is critical for designing much-needed therapies to decrease the morbidity and mortality associated with ALD and other disorders characterized by a vitamin A deficiency.

## 4. Materials and Methods

### 4.1. Mice and Treatments

All animal experiments and protocols were approved by the Institutional Animal Care and Use Committees (IACUC) of the WCMC. We generated conditional knockout (KO) transgenic female and male mice by crossing albumin Cre (AlbCre) mice (#005657; The Jackson Labs) with RARβ fl/fl mice (floxed in exons 9 and 10) on a C57Bl/6 background [20] to obtain liver-specific RARβ knockout mice (BKO), which exhibited an almost complete elimination of the ligand-binding domain (LBD) of RARβ, resulting in its disruption (Supplementary Figure S1). To mimic the early stages of alcohol-associated liver disease (ALD), we treated mice with the Lieber–DeCarli diet for 3 weeks (Supplementary Figure S2A and Supplementary Materials and Methods). We followed a previously published protocol [33] with some changes.

### 4.2. Triglyceride Measurements

We performed triglyceride extraction in frozen liver samples (50 mg tissue) using the Folch method and the Infinity triglyceride reagent (TR22421, Invitrogen, Waltham, MA, USA), as previously described [45].

### 4.3. Blood Alcohol Level Measurements

To measure blood alcohol levels, we used an ethanol assay kit (MAK076; Sigma, St. Louis, MO, USA) on mouse plasma collected at the time of sacrifice.

### 4.4. RNA Isolation and qRT-PCR

We isolated total RNA from the livers of the WT and BKO mice fed the ETOH diet (n = 5 for each genotype) and pair-fed (n = 4 for each genotype) using the RNeasy Mini Kit (74104, Qiagen, Hilden, Germany) with in-column DNAse I treatment (1023460, Qiagen), which was followed by NanoDrop quantification, reverse transcription, and qRT-PCR (Quanta Biosciences, Beverly, MD, USA) (Supplementary Materials and Methods and Supplementary Table S1 for the list of primers used).

### 4.5. Southern Blotting and Immunostaining

To determine the genotypes of the RARβ knockout mice we performed Southern blot analysis (as detailed in Supplementary Figure S1 and the Supplementary Materials and Methods). To measure the levels of specific proteins we performed immunohistochemistry and western blotting (Supplementary Table S2 for the list of antibodies used).

## Data Availability

The data are available upon reasonable request.

## Supplementary Materials

The supporting information can be downloaded at: https://www.mdpi.com/article/10.3390/ijms241512035/s1. References [46,47,48,49] are cited in Supplementary Materials.
