# Supplementary material for: Retinoic Acid Receptor β Loss in Hepatocytes Increases Steatosis and Elevates the Integrated Stress Response in Alcohol-Associated Liver Disease
Source: Int J Mol Sci. 2023 Jul 27;24(15):12035. doi: 10.3390/ijms241512035 (PMC10418449; doi:10.3390/ijms241512035)
Supplement: Supplementary file 1 [file ijms-24-12035-s001.zip › ijms-2482914-supplementary/ijms-2489214 Suppementary Table S1.pdf]

Table S1. List of primers.

Gene ID	Forward (5'-3')	Reverse (5'-3')	Amplicon size (bp)
Acadm	AGGAGCCCGGATTAGGGTTT	TTGTCATATTCCGGGGCGAC	115
Acc1	ATGGGCGGAATGGTCTCTTTC	TGGGGACCTTGTCTTCATCAT	148
Acsl1	GTCCTGGGCACAGAAGAGAG	GTCAGAAGGCCGTTGTCAAT	186
Angptl4	GCCCAAGGGAAAAGATGCAC	GGGCCACCTTCTGGAACAAT	140
Asns	TGGGTTTCTGGCTGTGTGTT	TGGGAAGAGTTTCTCCACGC	217
Atf4	ATGGCCGGCTATGGATGATG	TCCAACGTGGTCAAGAGCTC	174
Atf5	CTGGCTCGTAGACTATGGGA	CCCGCTCAGTCATCCAATCA	146
Cd36	CTATTGGCCAAGCTATTGCG	TCAGATCCGAACACAGCGTA	135
Cpt1a	GTCCTGCAACTTTGTGCTGG	CAAACAGTTCCACCTGCTGC	260
Cpt1b	ATGGCATCCTTAGGGGTGTG	ATGCAATCGACCAGTCCCAT	118
Cyp4A10	GCTCAAGACCCTCCAGCATT	CTGTAAGCAGGCACTTGGGA	124
Cyp4A14	TGCAGAAGGCCAGGAAGAAG	GGGTGGCCAGAGCATAGAAA	180
Cyp4A31	ATCACCGCCCTTTCACTGG	AAACCATACCCTGATCGCCC	189
Cyp7A1	CACCATTCCTGCAACCTTCTGG	ATGGCATTCCCTCCAGAGCTGA	132
Fasn	GGAGGTGGTGATAGCCGGTAT	TGGGTAATCCATAGAGCCCAG	140
Fatp1 (Slc27A1)	GAAAAAGGATGCCGTGTCCG	CTTCAGACCTCCACGACTCC	125
Fgf21	GTGTCAAAGCCTCTAGGTTTCTT	GGTACACATTGTAACCGTCCTC	123
Gdf15	ATGCACAGGACAGACAGTGG	CCAGCCCGACCCCAATAAAT	168
Gsta1a	AGTGCCCATGGTGGAGATTG	CCTGTTGCCCACAAGGTAGT	295
Gsta1b	AAGAGCTTGATGCCAGCCTT	CCAAGGGAGGCTTTCTCTGG	123
Hmgcs2	CAGCCCAGCAGAGGTTTTCTA	CTCCTGGACTGAACAGAAGCC	209
Hmox1	ACAGCCCCACCAAGTTCAAA	AAGTGACGCCATCTGTGAGG	191
Nqo1	CATTGCAGTGGTTTGGGGTG	GAGTACATGGAGCCGCTACC	163
Nrf2	ACTACAGTCCCAGCAGGACAT	CCTTCTGGAGTTGCTCTTGT	164
Pck2	CAACCAGAGGGCATCCACAT	TTTCTACCCGTGCCACATCC	154
Pgc1a	GAATCAAGCCACTACAGACACCG	CATCCCTCTTGAGCCTTTCGTG	136
Plin5	CGGGTCTGTCCCTACTGGAA	CGATTCACCACATTCTGCTGG	139
Sorbs1	CCTCGTCTTCCTACAGGGGA	GTCAGCAGAAAGGCTGGGAT	184
Trib3	CTCTTCCGGCAGATGGCTAG	GCAGGCATCTTCCAGGTTCT	138
RARβ	ACCAGATCACCCTGCTCAAA	GTCAGTCAGAGGACCGAAGC	155
36B4	AGAACAACCCAGCTCTGGAGAAA	ACACCCTCCAGAAAGCGAGAGT	448
Xbp1	GAACCAGGAGTTAAGAACACG	AGGCAACAGTGTCAGAGTCC	205 (unspliced);<br>179 (spliced)
