# Supplementary material for: Retinoic Acid Receptor β Loss in Hepatocytes Increases Steatosis and Elevates the Integrated Stress Response in Alcohol-Associated Liver Disease
Source: Int J Mol Sci. 2023 Jul 27;24(15):12035. doi: 10.3390/ijms241512035 (PMC10418449; doi:10.3390/ijms241512035)
Supplement: Supplementary file 1 [file ijms-24-12035-s001.zip › ijms-2482914-supplementary/ijms-2489214 Supplementary Materials and Methods.pdf]

Supplementary Materials and Methods

Mice and Treatments. All animal experiments and protocols were approved by the Institutional Animal Care and Use Committees (IACUC) of WCMC. We generated conditional knockout (KO) transgenic female and male mice by crossing albumin Cre (AlbCre) mice (#005657; The Jackson Labs) with RARβ fl/fl mice (floxed in exons 9 and 10) (1) on a C57Bl/6 background to obtain hepatocyte-specific RARβ knockout mice (BKO), which exhibit an almost complete elimination of the ligand binding domain (LBD) of RARβ resulting in its disruption. To assess the elimination of the LBD portion of RARβ we performed southern blotting, as detailed in the section below (Southern Blotting) and in **Supplementary Figure S1**. For the ethanol treatments in mice, at 2 months of age, we randomly placed 2 mice per cage and had them adjust to the Lieber DeCarli control liquid diet (F1258SP, Bio Serv) for 3 days as previously reported (2). This period of time is known as acclimatization period and precedes the incremental concentrations of ETOH feeding, as outlined in **Supplementary Figure S2A**. After the 3 days of acclimatization, we divided the mice in pair-fed-treated (PF) and ETOH-treated (ET) groups in wild type (WT) female mice (PF, n = 9; ET, n = 12) and BKO (PF, n = 4; ET, n = 6). The diets were prepared daily and were provided at 5:30 PM, which is when mice start to be more active. We provided the lowest concentration of ETOH Lieber DeCarli diet (F1258SP, Bio Serv) (1% v/v) on days 4-5, followed by 2% on days 6-7, 4% on days 8-10, 5% on days 11-16, and finally, 6.4% on days 17-21 (**Supplementary Figure S2A**). The control and ETOH diets are isocaloric to ensure that the differences between ETOH-fed and control diet-fed mice are due to ETOH alone and not because of differences in the amount of calories in the diet. Our feeding

protocol is similar to previously published methods (3) and recapitulates early stages of ALD and ETOH-induced hepatic injury. We sacrificed the mice by cervical dislocation in the morning because mice prefer to eat in the nocturnal hours, thus, ETOH blood levels peak at the time of sacrifice (2). We collected the liver and blood for biochemical and molecular experiments. For this protocol we recorded food consumption and body weight daily and reported it in **Supplementary Figure S2B**.

Histopathological evaluation. After sacrifice, a liver pathologist at New York-Presbyterian Hospital/Weill Cornell Medical College evaluated each hematoxylin and eosin (H&E)-stained section according to previously established criteria (4). The results of the histopathologic analysis are reported in **Figure 1B**. The pathologist was blinded to the identity of the experimental groups.

Southern Blotting. To determine the genotype of the RARβ knockout mice (BKO) we performed a southern blot analysis (**Supplementary Figure S1**). We isolated genomic DNA from frozen liver by lysing the tail tissue in lysis buffer (containing 50 mM Tris, 100 mM EDTA, 0.5% SDS, and 1 mg/mL Proteinase K) at 55 C for 16 hours followed by a phenol chloroform extraction, as previously described (5). After resuspending the DNA pellet in TE buffer, we digested 8 ug of DNA with 20 U of NheI (NEB #R3131) in the supplied buffer at 37 C for 16 hours and then fractioned it on an 0.8% agarose gel in 1X TAE. When the gel finished running, we denatured the DNA by incubating the gel in a solution containing 2% NaOH and 1.5 M NaCl for 30 minutes at room temperature. We transferred the DNA to a nitrocellulose membrane in 20X SCC overnight. The following morning, we irradiated the membrane with 120,000 microjoules/cm2 in a stratalinker to crosslink the DNA to the membrane before

blocking the membrane with 1 mg of salmon sperm DNA in a final volume of 10 ml pre hybridization solution (containing 50% v/v formamide, 5X SCC, 50 mM phosphate buffer, 5 mM EDTA, 10 Denhardt's solution, 0.1% SDS, 10% w/v dextran sulfate) for 5 hours at 42 C. We next added a labeled cDNA probe containing E1 of the RARβ allele. We labeled the cDNA probe with random priming using [32P]dCTP and used 20,000,000 cpm for the hybridization. After 20 hours of hybridization, the membrane was washed twice for 20 minutes per wash in a sodium chloride and sodium citrate solution (low stringency solution) of at 42 C and three times for 5 minutes per wash in a sodium chloride and sodium citrate solution (high stringency solution) at 55 C. The membrane was exposed to a phosphor screen for 24 hours before acquiring the image on a Typhoon FLA 9500 phosphor imager. The image of a representative experiment is shown in Supplementary Figure S1C.

Cell Cultures and Treatments. We purchased the human hepatoma cell line HepG2 from the American Type Culture Collection (HB-8065, ATCC) and cultured these cells in Dulbecco's modified Eagle's medium (DMEM) supplemented with 10% heat-inactivated fetal bovine serum (FBS). In the same medium we also cultured cells from the recently generated RARβ knockout (KO) HepG2 cell line.

We used the mouse hepatocyte cell line AML12 (ATCC, CRL-2254) used as a representative nontransformed mouse hepatocyte cell line, and cultured these cells in DMEM + 10% FBS (S10250, R&D Systems), 10 µg/ml insulin, 5.5 µg/ml transferrin, 5 ng/ml selenium (iTX mix 354352, Corning), 40 ng/ml dexamethasone (D4902, Sigma), and 7 ng/ml glucagon (G2044, Sigma). For cell counts we used a cell counter (Z1 Particle Counter; Beckman-Coulter). All ETOH treatments were performed using the

appropriate media used for each cell line unless specified, with the addition of HEPES at a 20 mM concentration.

We treated the cells with ETOH at a concentration of 100 mM for 0, 24, and 72 hours (h) in tightly closed T25 flasks, as shown in the **Figure 4** diagram, to avoid ETOH evaporation, as previously reported and harvested the cells 5 hours from the last ETOH treatment. The addition of HEPES in the media avoided ph changes in sealed flasks over the course of the experiments. To harvest the cells, we washed 3 times on 1X PBS followed by scraping of the cells, centrifugation to remove the 1X PBS, and lysis of the pellets with final sample buffer (described in the western blotting section) for western blotting or lysis with Trizol for RNA isolation.

Crispr/Cas9 Technology. To delete one portion of the ATF4 gene in parental and RARβ knockout KO HepG2 cell lines, we used the Synthego online tool (Synthego; Redwood City, CA) to design guide RNA sequences. The gRNA sequences that gave us the best KO efficiency were A*G*A UGACCUUCUGACCACGU and U*A*A UAAGCAGCCCCCCCAGA located in exon 2 of the ATF4 sequence. After reconstituting the gRNA sequences as we previously published (6), we plated 50,000 cells in 24-well plates and after they attached, we treated them with the gRNA/Cas9 mix for 24h. To determine the KO efficiency, we performed Sanger sequencing on the genomic DNA on single cell clones compared with an unedited reference genome using the online tool provided by Synthego, Inference of CRISPR Edits (ICE). Along with the Synthego ICE tool, we assessed the KO efficiency by performing ATF4 western blotting (**Figure 6**).

RNA Isolation and qRT-PCR. In liver tissues, we isolated total RNA of WT and BKO mice fed with the ETOH diet (n = 5 for each genotype) and pair-fed (n = 4 for each genotype) using the RNeasy Mini Kit (74104, Qiagen) with in-column DNAse I treatment (1023460, Qiagen) followed by Nanodrop quantification and reverse transcription (Quanta Biosciences, Beverly, MD). The cDNA was then diluted 1:10 with water. For real-time PCR reactions, we used 3 μl of cDNA mixed with 0.4 μM primers and 7.5 μl SYBR Green Supermix (Quanta Biosciences) in a 15 μl reaction. Primer sequences are listed in **Supplementary Table S1**. The reactions were run on a MyiQTM Single-Color Real-Time PCR Detection System (Bio-Rad). To calculate the transcript levels in the experimental groups we used the delta Ct method and normalized to the mRNA levels of the mouse internal control gene 36B4 (7).

RNA Sequencing (RNA-Seq). We isolated total RNA from livers of mice fed with the ETOH diet (n = 3) and pair-fed (n = 3) followed by Nanodrop quantification and Bioanalyzer quality assessment. Library preparation was performed in the Weill Cornell Genomic Core Facility using the TrSeq RNA protocol (Illumina) as previously described (3, 6). Briefly, we generated 250 – 300 bp cDNA fragments that were PCR-amplified and treated with RNaseH to digest residual mRNA strands. After ligation to the pair end adaptors, we purified the cDNA and performed 15 PCR cycles before sequencing.

Sequencing and Data Analysis. We sequenced the libraries with paired-end 51 bps on the Illumina NovaSeq6000 following previously published methods (6). The sequencing reads were aligned to the reference mouse genome (GRCm38 assembly) using STAR v2.5.2b. Read counts were calculated using HTseq-count v0.11.2 (8) and

differential expression analysis was performed using DESeq2 v1.26. P-values were corrected using the Benjamini-Hochberg method for multiple testing. The RNA-seq data accession number is GSE221713.

Western Blotting. At the end of the treatments, we collected the media and washed the cells in cold 1X PBS for 3 times. Next, we scraped the cells and centrifuged them for 5 minutes at 3000 rpm to remove the 1X PBS. Next, we lysed the cell pellets in final sample buffer (0.125M Tris-HCl, pH 6.8, 2% SDS, 2.5% beta-mercaptoethanol) according to previously reported methods (3, 6) and boiled the lysates for 5 minutes to denature the proteins. We performed protein quantification with the RC DC Protein Assay kit (Bio Rad) followed by loading 30 µg of protein input in 10 to 12% sodium dodecyl sulfate–polyacrylamide gels. Next, we transferred the proteins in nitrocellulose membranes (162-0115, Bio-Rad). After blocking in 5% milk dissolved in TBS-0.2% tween for 1h, we incubated the membranes with the antibodies listed in **Supplementary Table S2** overnight at 4°C. Next, we washed the excess of primary antibody 3 times with TBS-t for 5 minutes each wash, followed by incubation with the appropriate secondary antibody (Jackson Labs) for 1h at room temperature. To develop the signal we used the ECL Blotting Substrate (32106; Thermo Scientific). We quantified the western blottings with Fiji (ImageJ software) by calculating the ratio between the protein of interest and the appropriate loading control.

Immunohistochemistry. We performed immunohistochemistry in paraffin-embedded 5 µm-thick sections following already published methods (9). After re-hydration of the sections in gradually decreasing alcohol concentrations, we performed heat-induced antigen retrieval using either the citrate-based (Cat# H-3300; Vector Laboratories) or

the tris-based (Cat# H-3300; Vector Laboratories) antigen unmasking solution according to the manufacturer's recommendation. After 10 minutes in a 3% hydrogen peroxide solution in methanol, we blocked the tissues with 10% goat serum in 1X PBS for 1 hour. Next, we incubated the slides with the primary antibodies overnight at 4°C. On the next day we incubated the slides for 1 hour with the secondary antibody 1X goat anti-rabbit IgG (B40962; Invitrogen). As a negative control, one of the two sections in each slide was incubated in parallel without primary antibody. Positive signal was developed with 3,3'-diaminobenzidine (DAB). We acquired >5 fields per section with a Nikon TE2000 microscope. For quantification of the DAB-positive signal, we averaged the % area positive per field with the Fiji (Image J) software as previously reported (3).

Measurement of Reactive Oxygen Species (ROS). To measure the levels of oxidative stress as a result of oxygen reactive species (ROS) production caused by ETOH, we stained live parental and RARβ KO HepG2 cells with CellROX green reagent (#C10444; Invitrogen) after treatment with ETOH. CellROX green emission fluorescence is at 520 nm. We incubated the cells at a concentration of 2.5 μM in culture media for 40 minutes in the incubator. To stain the nuclei, we used NucBlue (#R37605; Invitrogen), which labels the cell nuclei in blue, at the concentration suggested by the manufacturer. We acquired a minimum of 6 fields per experimental group with a Nikon TE2000 fluorescence microscope and quantified the level of fluorescence with Fiji (Image J) normalizing by the number of nuclei per field. These experiments were performed 3 times independently.

References

1. Chapellier B, Mark M, Bastien J, Dierich A, LeMeur M, Chambon P, Ghyselinck NB. A conditional floxed (loxP-flanked) allele for the retinoic acid receptor beta (RARbeta) gene. Genesis 2002;32:91-94.
2. Bertola A, Mathews S, Ki SH, Wang H, Gao B. Mouse model of chronic and binge ethanol feeding (the NIAAA model). Nat Protoc 2013;8:627-637.
3. Melis M, Tang XH, Attarwala N, Chen Q, Prishker C, Qin L, Gross SS, et al. A retinoic acid receptor β2 agonist protects against alcohol liver disease and modulates hepatic expression of canonical retinoid metabolism genes. Biofactors 2021.
4. Brunt EM, Kleiner DE, Wilson LA, Belt P, Neuschwander-Tetri BA, (CRN) NCRN. Nonalcoholic fatty liver disease (NAFLD) activity score and the histopathologic diagnosis in NAFLD: distinct clinicopathologic meanings. Hepatology 2011;53:810-820.
5. Hosler BA, Rogers MB, Kozak CA, Gudas LJ. An octamer motif contributes to the expression of the retinoic acid-regulated zinc finger gene Rex-1 (Zfp-42) in F9 teratocarcinoma cells. Mol Cell Biol 1993;13:2919-2928.
6. Tang XH, Melis M, Lu C, Rappa A, Zhang T, Jessurun J, Gross SS, et al. A Retinoic Acid Receptor β2 Agonist Attenuates Transcriptome and Metabolome Changes Underlying Non-Alcohol-Associated Fatty Liver Disease. J Biol Chem 2021:101331.
7. Trasino SE, Tang XH, Jessurun J, Gudas LJ. A retinoic acid receptor β2 agonist reduces hepatic stellate cell activation in nonalcoholic fatty liver disease. J Mol Med (Berl) 2016;94:1143-1151.
8. Anders S, Pyl PT, Huber W. HTSeq--a Python framework to work with high-throughput sequencing data. Bioinformatics 2015;31:166-169.
9. Melis M, Tang XH, Trasino SE, Patel VM, Stummer DJ, Jessurun J, Gudas LJ. Effects of AM80 compared to AC261066 in a high fat diet mouse model of liver disease. PLoS One 2019;14:e0211071.
