# Supplementary material for: Retinoic Acid Receptor β Loss in Hepatocytes Increases Steatosis and Elevates the Integrated Stress Response in Alcohol-Associated Liver Disease
Source: Int J Mol Sci. 2023 Jul 27;24(15):12035. doi: 10.3390/ijms241512035 (PMC10418449; doi:10.3390/ijms241512035)
Supplement: Supplementary file 1 [file ijms-24-12035-s001.zip › ijms-2482914-supplementary/ijms-2489214 Supplementary Table S2.pdf]

Table S2. List of antibodies used.

Antibody	Species	Cat. N.	Company	Lot	Concentration
ATF4	Rb	1181S	Cell Signaling	5	WB 1:1000<br>IHC 1:100
4-HNE	Rb	ab46545	Abcam	NA	1:100
CYP2E1	Rb	NBP1-85367	Novus Biologicals	000003675	1:1000
NQO1	Rb	PA5-115666	Invitrogen	WI3372688A	WB 1:1000<br>IHC 1:100
ACTIN	Ms	AC004	AbClonal	3500100010	1:40000
HISTONE 3	Rb	06-755	Millipore	2615003	1:1000
BIP/GRP78/HSPA5	Rb	66574-1-IG	Proteintech	NA	1:5000
TNFa	Rb	A0277	AbClonal	5500004871	1:100
p-4EBP1 (T37/46)	Rb	9459	Cell Signaling	10	1:1000
4EBP1	Rb	9644	Cell Signaling	12	1:1000
