# Supplementary material for: Retinoic Acid Receptor β Loss in Hepatocytes Increases Steatosis and Elevates the Integrated Stress Response in Alcohol-Associated Liver Disease
Source: Int J Mol Sci. 2023 Jul 27;24(15):12035. doi: 10.3390/ijms241512035 (PMC10418449; doi:10.3390/ijms241512035)
Supplement: Supplementary file 1 [file ijms-24-12035-s001.zip › ijms-2482914-supplementary/ijms-2489214 Supplementary Tables and Figure Captions.pdf]

Supplementary Figure and Table Legends.

Supplementary Figure S1. Diagram of the Albumin Cre; RARβ flox/flox (BKO) Mouse Line. (A) Scheme of the RARβ exons (indicated as E) in which the loxp sites are located. Almost complete excision of the exons E9 and E11 (determining the loss of the ligand bind domain (LBD) of the RARβ gene) occurring in the progeny of the albumin Cre with the RARβ flox/flox mice. The dependence on the albumin promoter determines the RARβ liver knockout (BKO) mainly in hepatocytes. (B) Simplified schematic of the generation of the BKO mice. (C) Representative Southern blot indicating the shorter size (5.5 Kd) of the RARβ knockout gene. (D) RARβ assessed by qRT-PCR comparing levels between wild type (WT) and BKO mice. The RARβ mRNA levels were normalized to the mouse housekeeping gene 36B4 (n = 4 mice per group). The data are represented as mean ± standard deviation (SD). *= $p < 0.05$.

Supplementary Figure S2. Lieber DeCarli Diet, Diet Consumption, Blood Ethanol (ETOH) Concentration, and CYP2E1 Levels. (A) Timeline diagram of the Lieber DeCarli ethanol (ETOH) diet and matching isocaloric control diet. The ETOH concentration is indicated in % volume/volume (v/v). The duration of each ETOH feeding step is represented as days. Mice sacrifice (Sac) was performed at the 21st day of treatment. (B) Records of body weights (BW) and food consumption/BW ratio for wild type (WT) and BKO mice over the course of the 21 days of treatment represented. The pink area in each plot indicates the ETOH diet treatment. (C) Representative ETOH blood concentration for each experimental group (n = 3 mice per group) expressed as

mg/dL. Pair-fed (PF); ETOH-fed (ET). (D,E) Western blotting to assess the levels of CYP2E1 in ETOH-WT and ETOH-BKO mice and relative quantification calculating the ratio between the OD measurements of CYP2E1 with those of actin. Pair-fed (PF); ETOH-fed (ET). Gray bars indicate wild type mice (WT) whereas red bars indicate BKO mice. The data are represented as mean ± standard deviation (SD). *= $p < 0.05$; ***= $p < 0.001$.

Supplementary Figure S3. Immunostaining. Immunohistochemistry of the inflammation marker TNFα in the livers of AlbCre;RARβ knockout (BKO) and wild type (WT) mice. We performed immunostaining in representative liver sections of pair-fed (PF) mice (WT, n = 4; BKO, n = 4) and ETOH-fed mice (ET) (WT, n = 6; BKO, n = 6). As a negative control we used one slide incubated with the blocking buffer and the same secondary antibody as the rest of the sections. The quantification of the TNFα-positive cells is expressed as optical density % positive area. The data are represented as mean ± standard deviation (SD). Scale bar = 100 μm.

Supplementary Figure S4. Western Blotting for the ER Stress Protein BIP/GRP78. Western blotting of the endoplasmic reticulum (ER) stress-associated protein, BIP.GRP78 in wild type (WT) and BKO mice treated with a pair-fed (PF) control diet and ETOH diet (ET). Quantification performed calculating the ratio between the optical density (OD) measurements of BIP and the loading control histone 3 (H3). This membrane has been probed also for ATF4 in Figure 2, thus it shares the same loading control.

Supplementary Figure S5. Xbp1 Splicing in Wild Type and BKO Mice. Semiquantitative PCR of representative pair-fed (n = 4 mice per genotype) and ETOH-fed (n = 5 mice per genotype) mice liver samples. The ladder (L) is on the left side of the gel. The unspliced Xbp1 mRNA is observed at 205 base pair (bp), whereas the spliced variant is observed at 179 bp. As a positive control we used mouse embryonic cells (ESC) untereated (U) and after treatment with tunicamycin (4 ug/ml for 8 hours). We used 36B4 as housekeeping gene.

Supplementary Figure S6. Increased Oxidative Stress and ATF4 Network BKO and Wild Type Mice. Full images for the immunostaining performed to detect the oxidative stress marker 4-Hydroxynonenal (4-HNE), ATF4 and NQO1, in the livers of BKO and wild type (WT) mice shown in Fig. 3. Scale bar = 100 µm.

Supplementary File S1. RNA-Seq Statistically Significant Genes. The first sheet represents the list of genes that passed the statistical test, as described in Supplementary Materials and Methods. For RNA-seq we sequenced three pair-fed (PF) and three ethanol-fed (ETOH) wild type mice. The second sheet indicates the list of genes with a fold change (FC) higher than 1.5 in the ETOH-fed versus pair-fed mice.

Supplementary File S2. Enrichr Analysis Pathways. The first sheet indicates the pathways identified using Enrichr as explained in Supplementary Materials and Methods. Each database is highlighted in green, whereas the pathways associated with

oxidative stress are indicated in red. In the second sheet we show only the list of the pathways associated with oxidative stress.

Supplementary Table S1. List of the mouse genome primers used to perform qRT-PCR. The table indicates the gene ID, the forward and reverse primer sequences, and the amplicon size.

Supplementary Table S2. List of antibodies used to perform western blot and immunostaining.
